# Metal-on-Metal Hip Arthroplasty: A Comprehensive Review of the Current Literature

**DOI:** 10.7759/cureus.48238

**Published:** 2023-11-03

**Authors:** Timothy P Davis

**Affiliations:** 1 Department of Anatomy, University of Nottingham Medical School, Nottingham, GBR

**Keywords:** adverse reaction to metal debris, metal-on-metal total hips, total hip arthroplasty: tha, metal-on-metal, hip joint, primary arthroplasty

## Abstract

Metal-on-metal (MoM) total hip arthroplasty has been widely used since the end of the 20^th^ century, although rates have now decreased due to concerns regarding adverse reactions and failure rates. The MoM implant has been replaced with other materials, such as ceramic-on-ceramic (CoC) and metal-on-polyethylene (MoP). This literature review looks at the past and present use of MoM prostheses to assess whether the turn away from MoM use is justified. Online literature searches were performed on PubMed, Ovid Medical Literature Analysis and Retrieval System Online (MEDLINE), and Web of Science online databases using the search terms “MoM and (ARMD and ALVAL)” (ARMD: adverse reaction to metal debris; ALVAL: aseptic lymphocyte-dominant vasculitis-associated lesion). A total of 64 relevant titles were included in the review. Although risk factors for adverse reactions and the causes of ARMD are generally agreed upon, more work is required to further understand the specific thresholds of blood metal ion levels that can be used to consistently identify ARMD and excessive metal wear-debris in patients who have not had their MoM implants revised. Metal-on-metal devices are not an acceptable option for total hip arthroplasties (THAs) in their current formulation due to the high rate and risk of ARMD. Some MoM hip resurfacing operations are appropriate for very carefully selected patients who are fully aware of the risks posed by the implant. It is recommended that device-specific thresholds for metal ion levels be developed to identify patients at risk of ARMD locally and systemically while using auxiliary tools to assist diagnosis, such as metal artefact reduction sequences (MARS)-MRI and hip scoring tools. Further work should investigate device-specific blood metal ion levels, the systemic effects of raised metal ion concentrations secondary to MoM arthroprosthetic wear, and the potential risks of ARMD caused by wear from tapered stems (including the implications this has for patients with CoC and MoP prostheses).

## Introduction and background

The realm of orthopaedic surgery has witnessed several transformative interventions over the past century, with hip arthroplasty standing out as a particularly pivotal procedure [1]. Offering a renewed lease on life to countless individuals plagued by debilitating hip ailments such as, but not limited to, osteoarthritis, rheumatoid arthritis, fracture of the femoral neck, and ankylosing spondylitis, this surgical intervention promises both pain alleviation and the restoration of mobility [2]. Among the varied types of hip arthroplasties, the metal-on-metal (MoM) variant (usually made with a cobalt-chromium-molybdenum femoral ball) [3] emerged with great promise, capturing the attention of both medical professionals and patients alike [4].

Originally, MoM's allure stemmed from a combination of its potential benefits. These included reduced wear, given the hardness of the materials, and the possibility of a larger diameter head, which promised increased stability and reduced risk of dislocation [4-6]. Theoretically, these attributes made MoM especially attractive for younger and more active patients who needed durable solutions that could withstand increased physical demands.

However, as is often the case in the medical world, transitioning from laboratory findings and theoretical benefits to real-world applications unveiled a host of challenges. Not long after its widespread adoption, concerns began to surface. Medical professionals and researchers started documenting instances of adverse reactions and implant failure [7], some of which were severe and unexpected. Papers suggest that the primary cause of this was metal debris. As MoM prostheses function, they shed microscopic metal particles into the surrounding tissues [3]. These particles, in some patients, triggered a cascade of adverse reactions, collectively termed adverse reactions to metal debris (ARMD) [8]. The manifestations of ARMD can vary widely: locally in metallosis, inflammation, including aseptic lymphocyte-dominant vasculitis-associated lesions (ALVAL), pseudotumours, and necrosis, and systemically with potential organ toxicity, tumour genesis, and immunotoxicity. These local effects can manifest as asymptomatic tissue changes detectable only through imaging to severe local tissue damage causing pain and implant failure [9-15].

The medical and patient communities' initial enthusiasm for MoM was tempered by these revelations. The decreased rates of MoM procedures in recent times serve as a testament to the concerns associated with ARMD and other potential complications [3, 12, 16]. But, as with any medical dilemma, it's crucial to approach the MoM debate with nuance. While the concerns are valid and documented, there may be a subset of patients who would benefit from a MoM prosthesis. In embarking on this review, our goal is to provide clarity on the MoM conundrum from the available literature by discussing the historical and current consensus in relation to MoM prosthesis use in hip arthroplasty. We hope to inform medical practitioners and, therefore, patients, aiding them in making informed, evidence-based decisions.

Methods

This review started in November 2016. An online literature search was performed on the PubMed, Ovid Medical Literature Analysis and Retrieval System Online (MEDLINE), and Web of Science online databases using the search terms “MoM and (ARMD and ALVAL)”. PubMed returned 48 results, Ovid Medline returned 67, and Web of Science returned 43. Of the 158 results, 88 were duplicates. Of the remaining 70 results, 13 were rejected due to irrelevance, such as being non-human studies. Fifty-seven relevant titles were included. A further literature search was performed in 2023 with the same search term on PubMed. Seven further results were returned, which were deemed relevant and not duplicates.

**Figure 1:**
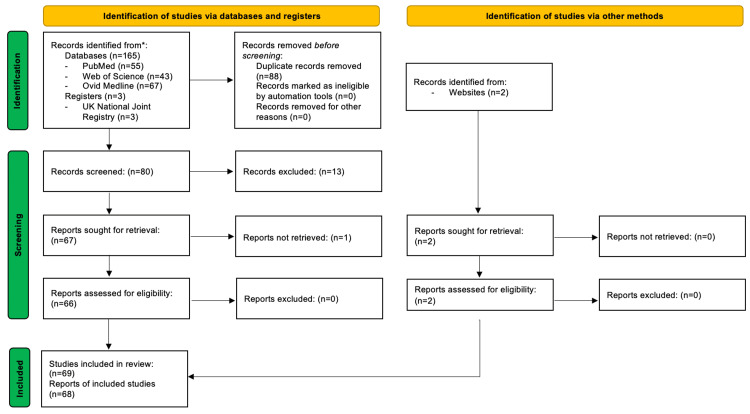
A PRISMA flow diagram demonstrating the methodology of study selection PRISMA: Preferred Reporting Items for Systematic Reviews and Meta-Analyses

The UK National Joint Registry (NJR) website, the UK National Institute for Health and Care Excellence (NICE) website, and the UK Medicines & Healthcare Products Regulatory Agency (MHRA) alerts pertaining to MoM hip implants were all used throughout the writing of the paper, thanks to their invaluable recommendations and reporting of outcomes with MoM implants. A flow diagram describing the above methodology is presented in Figure 1.

## Review

Discussion

Hip arthroplasty, an essential surgical procedure for addressing various hip joint pathologies, has advanced in recent times [1, 2]. The widespread use of MoM total hip arthroplasty at the end of the last century was driven by its purported advantages [3, 4]. However, like all medical innovations, real-world outcomes and complications often provide a clearer picture of the utility and safety of a particular approach.

One of the central concerns with MoM hip arthroplasty is the adverse reaction to metal debris. An ARMD is defined as aseptic “fibrosis, bone marrow oedema, tendon avulsions, and perivascular lymphocytic infiltration, seen as soft tissue or fluid masses adjacent to the hardware, also referred to as ALVAL [17]. It presents either symptomatically or asymptomatically, with symptoms including pain and an abnormal gait. It has been shown to not only affect soft tissue but also damage the bone (greater trochanteric fragmentation observed one to two years after MoM implantation) [18], therefore creating a wide range of symptoms and complications that surgeons must deal with, sometimes without any indication of the level of disease progression or even presence. An ALVAL was discussed by Willert et al. in 2005 [9], which was, at this point, seen as unusual: the most common inflammatory response seen in hip arthroplasty implant patients was macrophage-dominated (macrophages would infiltrate the tissue around the implant and engulf polyethylene debris; metal-on-polyethylene (MoP) was the most used implant material at the time). The immunological response seen by Willert et al. was lymphocyte-dominated and involved diffuse and perivascular chronic lymphocytic infiltrates with the development of high endothelial venules (HEVs). This was further illustrated by Davies et al. within the synovium [11]. Further analysis of chronic inflammation in MoM and MoP patients revealed that these cystic masses, or “perivascular lymphocytic cuffing,” were seen deeper, in the sub-synovium, in those patients who had MoM implants [19]. Both Davies et al. and Willert et al. described surface necrosis and the presence of metal debris but did not state the significance between these masses and vascular damage. Subsequent studies have defined the masses clinically as pseudotumours [10, 20, 21], formed secondary to the failure of MoM prostheses, and therefore categorised under the term ARMD. There seems to be a slight difference in ALVAL compared to other ARMDs: it is well recognised that ALVAL can occur in patients with very low wear states [22], thus it is likely that ALVAL is caused by a metal hypersensitivity reaction, while other ARMDs occur due to metal cytotoxicity.

A Finnish study concluded that there were two distinct types of inflammatory responses observed around pseudotumours [23]: especially high metal ion levels caused cytotoxicity and tissue injury; this led to the recruitment of macrophages and their removal of the necrotic debris (a macrophage-dominated response); histologically, it was like ‘polyethylene disease’ (polyethylene disease has been documented in those who have had metal-on-polyethylene hip prostheses and leads to implant loosening through local osteolysis) [3, 24]. The presence of metal particles within macrophages was observed. Lower metal ion levels were associated with T-lymphocyte-dominated responses and likely reflect a hypersensitivity reaction, like some people’s reaction to jewellery made from certain metals. This correlates with the aforementioned appearance of ALVAL in patients with very low wear states and associated low metal ion levels. The description of two distinct responses in failed MoMs is not even agreed upon, with a UK-based team identifying three different pathologies in 2014 [25]. They defined a macrophage subtype and a lymphocytic subtype, along with a subtype that was a mixture of both macrophages and lymphocytes. They did agree that the macrophage-dominated presentation was associated with high levels of wear debris. However, the lymphocyte-dominated presentation was not associated with low or high levels of wear debris but occurred equally in both environments (again indicating the likelihood of a hypersensitivity reaction), although it did infiltrate deeper into the local tissue than the macrophage response. Infiltration deeper into the tissue indicates smaller metal particle production, and this may be what stimulates the ALVAL response.

The risk factors for ARMD are poorly understood. However, it has been suggested that women are more susceptible to revision due to ARMD (Langton et al. document more complex local tissue reactions occurring in women with MoM hip replacements) [26]. In a study looking at hip resurfacing, the main risk factors for ARMD and failure of the prosthesis included smaller-diameter heads and poor alignment of the implant components [27]. There are differences in the risk factors for ARMD between total hip arthroplasty (THA) and hip resurfacing (HR): in HRs, an independent risk factor for revision is a head diameter smaller than 50mm [28] (backed up by the findings of the aforementioned Langton et al. paper in 2011 [27]), while in MoM THAs, a head larger than 50mm has been shown to be a risk factor for revision [7], a theory also suggested by Lombardi et al. [29]. Risk factors for pseudotumour formation in HR patients were investigated by Glyn-Jones et al. in 2009. Significant risk factors for revision were female gender (p<0.001), age under 40 years old (p=0.003), small components (p=0.003), and developmental dysplasia (p=0.019). These, however, were interrelated, and only gender (p=0.002) and age (p=0.024) had a significant effect on the revision rate after a Cox proportional hazard model was applied to the data [10]. It was speculated that patient weight might influence blood metal ion levels, with a heavy patient more likely to develop ARMD. This has since been discredited as it was found that there is no correlation between cobalt (Co) or chromium (Cr) blood ion levels and BMI [30] (Co and Cr were specifically tested for due to the Co-Cr alloy used in MoM prostheses).

Cobalt and chromium ions have been studied to determine the effects they have on the human body. It has been claimed that they promote the formation of certain cancers, but this has not been proven [31, 32]. Cobalt and Cr particles have been shown to be cytotoxic, with the ability to form reactive oxygen species and damage DNA and chromosomes when they are within cells (especially macrophages) [23]. In periprosthetic tissue, as already discussed, these can lead to osteolysis, inflammation, pain, and pseudotumours [3, 33, 34]. The full systemic effect these particles and ions have is unknown, and the pathogeneses of the adverse local tissue reactions they cause are largely a mystery, although some report local inflammation and fibrosis of minimal significance [27, 35], while others report cardiotoxicity and neurotoxicity, although these were in two individual case reports, respectively [36], and further literature has since urged caution in their findings due to the rarity of the findings and one of the case reports having been written by the author about themselves [37]. A further review of 12 years of National Joint Registry data, including 535,776 patients, found no increased risk of severe heart failure within seven years of operation in those who had undergone MoM arthroplasty compared to those who had undergone surgery with an alternative prosthetic used [38].

It would seem logical that increased wear would increase reported ARMD symptoms and that histological analysis would correlate with these symptoms. The causes of increased wear have been explored. Improper implantation of the prosthesis, specifically cups placed in greater abduction angles (>55 degrees) [39], or faulty product design can lead to increased wear on the wrong parts of hip implants, defined as ‘edge-loading.’ This ‘edge-loading’ process can occur in both THA and HR. A hip implant works as it is designed when the head of the implant and the acetabular component are oriented concentrically, with the acetabular component positioned in relation to the biochemical axis of loading and the contact patch (“the area of contact between the head and the acetabular component”). The weight of the load is shared between the lubricating synovial fluid and the solid metal articulating surfaces [40]. If any of the components of the hip implant move from this stable state, contact between the head and the acetabular rim can occur, and increased wear results. More wear debris production follows, and this theoretically leads to higher metal ion concentrations in the blood, hence the assumption that blood metal ion levels are a good indication of the state of MoM hip implant wear and potential local tissue and systemic reactions [3, 23, 41, 42]. This assumption, however, needs further research. Pijls et al. (2016) performed a meta-analysis that suggested that “the dose-response association of person/hip year exposure to MOM THA and/or levels of metal ions with the risk of mortality and other medical complications” needs to be further investigated [43]. This agrees with other studies: in one, 43% of patients with confirmed ARMD after revision had blood metal ion levels <7 ug/L (the proposed threshold for the diagnosis of ARMD: average population levels <2 ug/L), and another found that there was no correlation between pre-operative blood metal ion levels and the severity of the disease or outcome [44]. This indicates that the proposed threshold may not be appropriate to detect all ARMD cases and that blood metal ion thresholds should be lowered and then used with other tests and procedures to follow-up patients with MoM implants, a theory supported by several other studies [45, 46]. At odds with these findings, some studies do in fact conclude that blood metal ion levels are a reliable way to measure wear rate [26] and for identification of ARMD, but that these levels vary between implant designs, and therefore one threshold for all hip prostheses is not appropriate [47]. The amount of contradictory data available indicates that the risk of developing ARMD after implantation of MoM prosthesis is extremely variable, with factors such as surgeon experience, patient phenotype, and design and manufacture of the implant all playing a part.

Other tools include the Oxford Hip Score and the Harris Hip Score, developed in the late 90s and late 60s, respectively, to assess patient outcomes after hip arthroplasty. These measures, although not specifically designed for monitoring patients with potential ARMD, are useful in tracking patient progression or deterioration in relation to symptoms and function [48-50]. Magnetic resonance imaging has been suggested as an auxiliary tool in the diagnosis, staging, and surveillance of ARMD [14, 51, 52]. An MRI can be useful in characterising soft tissue disease where standard plain radiography may show limited disease or appear normal [53]. The UK MHRA recommends that all symptomatic individuals, all those with deteriorations in their Oxford Hip Score, and all patients with elevated or rising blood metal ions have a metal artefact reduction sequence (MARS)-MRI or US to delineate their disease prior to further intervention [54, 55].

While THA MoM may certainly seem like a less viable implant option when compared to current alternatives such as ceramic-on-ceramic (CoC) and MoP, especially for THAs, its utility in HR, particularly in young and active male patients with primary osteoarthritis, remains undeniable. Hip resurfacing is attractive as traditional THA design carries with it a significant risk of wear and failure, with often a technically challenging revision due to compromised acetabular and femoral bone stock; revision is more likely the younger a patient has a primary arthroplasty. Resurfacing offers these patients the opportunity to have an operation that conserves bone stock, reduces the risk of dislocation, and allows revision to a routine THA if or when required [56]. An ARMD and its associated conditions have been seen to occur in Birmingham HRs (BHR) [57], a specific HR model designed and manufactured by Smith & Nephew with excellent short- and long-term results reported from both designer and non-designer centres [54, 56, 58]. Surgeons are still implanting HRs, although their use has drastically declined over the last 10 years [59, 60]. This is likely due to the number of studies that advocate the use of BHRs [54, 58] and further support from national joint registry statistics thanks to widespread survival results concurrent with NICE guidelines [61], although it should be noted that some studies suggest that results are not reproducible between different surgeons [62]. Use of the BHR should be restricted to appropriately selected patients who are deemed to benefit from less-invasive surgery and maintenance of the femoral neck. Although ARMD has been reported in both MoM THA and HR, it is more prevalent in THA [63], theoretically due to the presence of stem tapers.

Experts have been puzzled in the past as to why revision due to ARMD occurs more frequently in THA patients than in HR patients [64]; now they believe that the answer lies in the debris arising from the taper junction [65]. Even though wear debris will occur at the MoM-bearing surface as the two articulating surfaces rub against each other, especially if misalignment occurs, it seems that the debris released from wearing at the taper junction is what causes the marked difference between MoM THA and HR. Indeed, this theory is supported by reports of ARMD in THAs with CoC and MoP wear surfaces [29, 66, 67]: metal debris production from the modular neck section of implants can be sufficient to cause ARMD, possibly answering the question of why there is an increased prevalence of ARMD in MoM THA compared to MoM HR. Modular THAs involve a two-part stem and neck. The stem module is implanted into the shaft of the femur, and the neck module is the equivalent of the neck of the femur. These are attached by a taper junction. The head of the implant is attached to the neck via a taper (this articulates with the acetabular component as previously). The head-acetabular interface could be CoC or CoP [67], but it doesn’t have to be MoM. The only MoM interface in the Vundelinckx et al. study was that of the stem-neck modular junction. Despite this being the only MoM interface, raised blood metal ion levels were identified in the study population, and one revision secondary to ARMD was reported in the 19 patients studied. Two studies have suggested that differences in manufacturing quality of the head taper within a single design have a large effect on wear: the rougher the head taper, the more wear the prosthesis undergoes [66, 68]. One of these studies has, however, been critiqued for its potentially inaccurate calculation of the roughness vs. waviness of the component [69]. Despite this critique, both studies show the need for a high level of vigilance when manufacturing medical devices, as an almost unnoticeable difference can lead to big variations in wear and potentially metal ion levels in vivo.

Looking at failure rates for MoM hips and the high prevalence of ARMD in MoM hip patients [29, 70, 71], it is easy to conclude that there is justification for the decline in the use of MoM hips, especially MoM THAs with a head diameter greater than or equal to 36 mm. Dislocation is not common in this size, but failure rates remain high, with 48% of these failures due to ARMD [29]. Another study of 36 mm heads showed 44% of revisions were ARMD-related, with 6.7% of the study population being revised at an average of 3.5 years (much higher than the NICE guidelines 5% revision rate at 10 years would recommend [61]. This high prevalence of ARMD is not restricted to large-head MoM THAs; it does occur in head sizes below 36mm in diameter [72].

Traditionally, younger patients have had less success with THAs [73, 74], but the BHR is still a safe and beneficial way for a distinct patient cohort to treat primary OA and maintain the femoral neck for future THA given its clinical results at 10 years, both with independent and designing surgeons [75-78]. The use of other HRs is questionable, and continued use should be based on clinical results at 10 years, both from independent and designing surgeons. The practice of HR is a lot more technically challenging than THA, and therefore surgeons carrying out clinical studies with HR prostheses must have sufficient experience implanting HRs [79]. To ensure ethically sound continued use, patients must be counselled on the intricacies of HR failure and the risks of MoM implantation, such as ARMD and associated conditions, so that they can make an informed decision about the type of implant they would like.

## Conclusions

In retrospect, the THA MoM episode in the journey of hip arthroplasty underscores the importance of vigilance, adaptability, and patient-centricity in medical practice. It serves as a testament to the need for ongoing research, feedback loops, and a commitment to learning from past experiences. As the field of hip arthroplasty continues to evolve, the lessons from the MoM chapter will undoubtedly influence future directions, ensuring safer and more effective outcomes for patients.

Metal-on-metal is not an acceptable option for THA prosthesis in its current formulation. Their survival statistics at 10 years are not adequate to justify their continued implantation. This is not to say that some HR operations are inappropriate in very carefully selected patients who are fully aware of the risks posed by the implant; they can, in limited circumstances, provide long-lasting, functionally improved hips that aid return to sporting activity.

Currently, more work is required in this area to further understand the specific thresholds of blood metal ion levels that can be used to consistently identify ARMD and excessive metal-wear debris in patients who have not had their MoM implants revised; patient follow-up is now of the utmost importance. It is essential that device-specific thresholds for metal ion levels are used to identify patients at risk of ARMD locally and systemically while using auxiliary tools to assist diagnosis, such as MARS-MRI and hip scoring tools, as recommended by national guidelines. Further work should also look at the systemic effects of raised metal ion concentrations secondary to MoM arthroprosthetic wear and the potential risks of ARMD caused by tapered stems, including the implications this has for patients with CoC and MoP prostheses.
